# Report of a Case of Jacksonian Epilepsy1Read at the thirtieth annual meeting of the Medical Association of Central New York, at Buffalo. October 19, 1897.

**Published:** 1898-04

**Authors:** F. H. Stephenson

**Affiliations:** Syracuse, N. Y.; Neurologist to Hospital for Women and Children


### REPORT OF A CASE OF JACKSONIAN EPILEPSY.[1]

BY F. H. STEPHENSON, M. D., Syracuse, N. Y.
Neurologist to Hospital for Women and Children.

MRS. B. came under my observation in June, 1886, suffering from very severe nocturnal headaches, these subsiding to a great extent early in the morning, leaving her head very sensitive to the slightest jar or noise. There was also marked tenderness of the entire scalp. The temperature was slightly elevated, registering 101°. The tongue was coated and there was an irregular rash on the chest and forehead, also a characteristic flat, scaly, dull colored syphilitic eruption on the inner side of the palms and wrists. Urinary analysis revealed no abnormality of the kidneys, nor was there constipation or other irregularities.

Mercurial inunction speedily controlled the headaches and other uncomfortable symptoms. A thorough course of medication, *i. e.*, mercurials, iodide of potassium and tonics, was continued at regular

1. Read at the thirtieth annual meeting of the Medical Association of Central New York, at Buffalo, October 19, 1897.

intervals during the following three years. The patient gained flesh and remained in good health during this period, although she was greatly worried and overtaxed, having nursed her husband who suffered with and died of general paresis during this interval.

In October, 1890, four years after infection, the patient had a severe attack of bronchitis, family and financial worries followed, causing headache, loss of sleep and a general bad condition at the time she applied for treatment; but she complained of nothing which in any way suggested a rekindling of her old trouble, though she had complained to her family of some pain and a numb feeling in the left arm; also a clutching, prickling sensation and numbness of the left calf. She did not mention these last symptoms to me, thinking they were not of importance.

On November 12th, at 11 A. M., she fell from her chair and had a general convulsion. This ceased in a few minutes, leaving her in a dazed condition. In forty-five minutes the left arm and hand began to be numb and contract, this spasm also affected the shoulder and the left side of the face, which was drawn upward. There was spasmodic action of the eyelids, the head was turned to the left and there was also jerking of the left limb. This convulsion continued, possibly, three or four minutes, when the spasm ceased, leaving the patient exhausted, but perfectly conscious. In fact, consciousness was not lost during any of the convulsions; the patient declared she knew all we said and did, but could give no indication of it. This, we know, is characteristic and diagnostic of Jacksonian epilepsy. Localised spasms of the left face, neck and arm occurred at intervals of about fifteen minutes during the next thirty hours, when they were followed by another general and severe convulsion; still another, though less severe seizure, occurred on the 15th, which was followed by some aphasia and left facial paralysis. Localised spasms recurred at increasing intervals until the 22d, though they became less marked and only momentary.

The pulse and temperature varied little from normal during this period. The evacuations were regular and the urine was normal. There was no anesthesia, hyperesthesia or tremor, although the left arm was much weaker and the grip less strong than normal. There had been no severe continued headache, vomiting, nausea, optic neuritis, or mental symptoms indicative of tubercle gumma, abscess, softening or exostosis, nor history of any injury or other organic derangement.

With the symptoms given and with a history of syphilis, I concluded there must have been a deposit sufficient to cause an irritant on, or in the cortical substance of the brain on the right side and involving the following motor centers: The lower two-thirds of the anterior and lateral central convolution on either side of the fissure of Rolando, the motor center for the face ; the middle third of the same convolution, the motor center for the shoulder, arm and hands. The spasmodic action of the lower extremity being so slight, I concluded the upper part of the convolution was very slightly involved in this possible meningitis, or overlapped by the deposit, although the first, or signal symptoms appeared in the leg. I refer, of course, to the area of the deposit.

The paralysis and motor aphasia passed away after a few days' treatment; these facts showed there was no organic change or thrombus in the brain. The aphasia was due probably to fluctuations of blood pressure or possibly to another slight focus of irritation. The fact of her having a few general convulsions would suggest the probability of a hemorrhage, but as no paralysis followed, only a weakness of the arm, and as the seizures were soon controlled, this idea was dissipated and my conclusion was that they were due to shock.

The convulsions were partially controlled by ether and enema, containing potassium bromide and morphia, in large doses, to lessen reflex irritability. Twenty drops of saturated solution of iodide of potassium were given at once, increasing the dose five grains every four hours until one hundred grains were taken. Each alternate dose was doubled and given in an enema, thus saving the stomach the severe distress and irritation which might follow. Inunctions of mercurial ointment of two drachms each were given on the softer surfaces of the body. As soon as chemico-physiological effects were obtained from these remedies, spasmodic action lessened and, as reported, finally entirely ceased. Some headache and numbness of the left arm and leg recurred until February 1st, after which date there were no symptoms to indicate the ordeal through which she had passed. Treatment was continued, taking alternately the iodide in increasing doses, then diminishing and using the mercurial inunction and tonics, thus effecting apparently a complete absorption of this deposit.

The patient remained well until March, 1896, ten years after infection, when some severe head pains accompanied by numbness, twitching and a grasping sensation of the left calf muscles caused her to report again for treatment. The old regime was carried out for six months, though all signal symptoms ceased after two weeks' treatment. In March, 1897, there was a repetition of the symptoms just given, suggesting increased cortical irritation, though after all of these years we have no symptoms pointing to serious organic change. *Doubtless* the epileptic attacks would develop were our treatment otherwise than vigorous at the outset.

We would hardly resort to surgical measures in such a case, although the point of irritation is fairly well known, because of the prompt control of the symptoms by internal remedies, and from the fact of there being a complete remission of all former symptoms and no constitutional or psychical disturbances.

This complete remission of symptoms is characteristic of syphilis and is an interesting factor of that disease. So long a period of central irritation, ten years, had it been otherwise than of specific origin, which yielded to treatment, would doubtless have developed serious inroads into the brain, causing death or imbecility. Sometimes partial convulsions, or Jacksonian seizures become general convulsions (incidentally, let me say Jacksonian seizures are caused by uremia), but irritation of the motor centers from the various growths, deposits or meningo-encephalitis are the most frequent causes of this form of epilepsy.

312 WARREN STREET.

